# Discovery of the *Alternaria* mycotoxins alterperylenol and altertoxin I as novel immunosuppressive and antiestrogenic compounds in vitro

**DOI:** 10.1007/s00204-024-03877-1

**Published:** 2024-10-02

**Authors:** Francesco Crudo, Vanessa Partsch, Dennis Braga, Ruzica Blažević, Judith M. Rollinger, Elisabeth Varga, Doris Marko

**Affiliations:** 1 Department of Food Chemistry and Toxicology, Faculty of Chemistry, University of Vienna, Währinger Str. 38, 1090 Vienna, Austria; 2 Doctoral School in Chemistry, Faculty of Chemistry, University of Vienna, Vienna, Austria; 3 Division of Pharmacognosy, Department of Pharmaceutical Sciences, University of Vienna, Vienna, Austria; 4 Unit Food Hygiene and Technology, Centre for Food Science and Veterinary Public Health, Clinical Department for Farm Animals and Food System Science, University of Veterinary Medicine, Vienna, Vienna, Austria

**Keywords:** In vitro toxicity, Chemical mixture, Food contaminants, *Alternaria* mycotoxins

## Abstract

*Alternaria* mycotoxins may pose significant challenges to food safety and public health due to the wide spectrum of reported adverse effects. Despite this, critical information on the immunomodulatory and antiestrogenic properties of most of these contaminants is still lacking. The present study aimed to identify the mycotoxins responsible for the immunosuppressive and antiestrogenic effects of a complex extract of *Alternaria* mycotoxins (CE) obtained by growing an *Alternaria alternata* strain on rice. Through a toxicity-guided fractionation procedure involving the production of CE-fractions by supercritical fluid chromatography and mycotoxin quantification by LC–MS/MS, the mycotoxins alternariol (AOH), tenuazonic acid (TeA), altertoxin I (ATX-I), and alterperylenol (ALTP) were identified as potential toxicologically relevant constituents contributing to the in vitro effects exerted by the extract. The assessment of the immunomodulatory effects, performed by applying the NF-κB reporter gene assay in THP1-Lucia™ monocytes, revealed the scarce contribution of AOH to the effects exerted by the CE. TeA showed no effect on the NF-κB pathway up to 250 µM, whereas ATX-I and ALTP suppressed the LPS-mediated pathway activation at concentrations ≥ 1 µM. The evaluation of antiestrogenic effects, performed in Ishikawa cells by applying the alkaline phosphatase assay, revealed the ability of ALTP (≥ 0.4 µM) and ATX-I (≥ 2 µM) to suppress the estrogen-dependent expression of enzyme activity. Given the risk of detrimental impacts stemming from alterations in endocrine and systemic immune responses by the investigated mycotoxins, further studies are needed to elucidate their underlying mechanisms of action and comprehensively evaluate the health risks posed by these toxins.

**Supplementary Information:**

The online version contains supplementary material available at 10.1007/s00204-024-03877-1.

## Introduction

Species of the genus *Alternaria* are known for producing mixtures of structurally diverse mycotoxins, some of which might pose a potential threat to human health due to their frequent occurrence in food and the wide array of toxic effects observed in vitro and in vivo (Ostry 2008; Escrivá et al. 2017; Crudo et al. 2019)*.* Despite over 70 *Alternaria* toxins being isolated and chemically characterized, toxicological and occurrence data are available for only a few of them. However, even for the most studied *Alternaria* mycotoxins, the available data are still not sufficient to allow a proper risk assessment and, consequently, for the setting of maximum levels in food (EFSA 2011, EFSA et al., 2016). In fact, in 2011, the European Food Safety Agency conducted only a preliminary evaluation of the risk of four *Alternaria* mycotoxins, namely alternariol (AOH), alternariol monomethyl ether (AME), tenuazonic acid (TeA), and tentoxin (TEN) (EFSA 2011). Notably, the estimated chronic dietary exposure to AOH and AME, which are known to exert genotoxic effects (Fleck et al. 2016; Hessel-Pras et al. 2019), was found to exceed the threshold of toxicological concern of 2.5 ng/kg b.w. per day, highlighting the potential threat posed by these mycotoxins to human health (EFSA 2011). Among the plethora of mycotoxins produced by this genus of molds, the epoxide-carrying perylene quinones altertoxin II (ATX-II) and stemphyltoxin III (STTX-III) have been reported to be even more genotoxic than AOH (Fleck et al. 2016), while very little is known about the mycotoxins altenuene (ALT), altertoxin I (ATX-I), alterperylenol (ALTP; also known as alteichin), and altersetin (AST). While genotoxicity and mutagenicity are frequently studied endpoints for certain *Alternaria* mycotoxins, research on the effects of this class of food contaminants on the immune and endocrine systems is limited, with the available studies mainly focusing on a few selected mycotoxins (Louro et al. 2024). Among the most studied mycotoxins, AOH and AME were reported to exert estrogenic effects in the Ishikawa cell line (Lehmann et al. 2006; Dellafiora et al. 2018), with AOH being active in a wide variety of different cell lines (Frizzell et al. 2013; Demaegdt et al. 2016; Stypuła-Trębas et al. 2017) and showing higher affinity for the estrogen receptor (ER) β than ERα (Lehmann et al. 2006). Both mycotoxins were also reported to negatively affect progesterone synthesis in vitro, while AOH was additionally shown to act as a full androgen agonist (Tiemann et al. 2009; Stypuła-Trębas et al. 2017). With regards to immune responses, AOH was found to partially inhibit the differentiation of human THP-1 monocytes into macrophages, to induce morphological changes in murine and human macrophages, and to alter the levels of pro- and anti-inflammatory cytokines at the mRNA and/or protein level (Solhaug et al. 2015, 2016; Grover and Lawrence 2017; Louro et al. 2024). In this context, one of the most important pathways involved in the immune responses is the NF-κB pathway, whose activation is also crucial for the regulation of inflammation and cell survival (Biswas and Bagchi 2016). Of note, AOH was reported to suppress the lipopolysaccharide (LPS)-induced activation of the NF-κB pathway in THP1-Lucia™-derived macrophages at concentrations ≥ 1 µM, and to enhance the levels of the anti-inflammatory cytokine IL-10 while reducing those of the pro-inflammatory cytokines TNFα, IL-8, IL-6 (Kollarova et al. 2018). Similarly, the genotoxic mycotoxin ATX II exhibited suppressive effects on the NF-κB pathway in THP1-Lucia™ NF-κB monocytes, while it failed to inhibit the nuclear translocation of the NF-κB subunit p65 in THP1-derived macrophages (Del Favero et al. 2020). Albeit with lower potency than AOH, suppression of pro-inflammatory cytokines was also reported for the mycotoxin AME in LPS-stimulated BEAS-2B cells (Grover and Lawrence 2017). Beyond the mycotoxins mentioned before, there is a shortage of in vitro data on the immunotoxic and (anti)estrogenic effects of the other *Alternaria* mycotoxins, as well as on the effects exerted by mycotoxins in mixtures. In this context, *Alternaria* species are known to produce mixtures of mycotoxins, whose presence in food raises concerns due to the potential for combinatory effects. Among the few studies investigating the impact of mycotoxin mixtures, Aichinger et al. (2019) reported the ability of a complex extract of *Alternaria* mycotoxins (CE), obtained by growing a strain of *Alternaria alternata* on long rice and comprising eleven chemically characterized compounds, to induce antiestrogenic effects in the endometrial Ishikawa cell line. These effects were observed despite the presence of the known estrogenic mycotoxins AOH and AME, suggesting that the CE might contain known and yet unknown compounds able to suppress the ER pathway. With respect to the immunomodulatory properties of the CE, no information is currently available. However, the presence of the immunosuppressive mycotoxins AOH and ATXII in the extract raises the question of whether the CE might potentially suppress the pathway despite the concentrations of AOH and ATX-II being much lower compared to those previously reported to induce immunosuppressive effects (Kollarova et al. 2018; Del Favero et al. 2020). Additionally, it prompts inquiry into the possibility of other immunosuppressive mycotoxins being present in the CE. Given these considerations and the potential health implications of the ingestion of foods contaminated with these ubiquitous mycotoxins, the objectives of the present study were to: i) identify the mycotoxins responsible for the antiestrogenic effects of the CE; ii) assess the immunomodulatory properties of the CE and identify the compounds involved in the effects observed. For this purpose, a toxicity-guided fractionation procedure, involving the production of various CE-fractions by supercritical fluid chromatography (SFC) and quantification of mycotoxins by liquid chromatography-tandem mass spectrometry (LC–MS/MS) analysis, was applied.

## Materials and methods

### Materials

Cell culture media and supplements used for culturing Ishikawa cells and THP1-Lucia™ NF-κB monocytes were purchased from Gibco® Life Technologies (Karlsruhe, Germany), except for charcoal–dextran stripped (CD-) FBS which was acquired from Fisher Scientific (Catalog #A3382101). 17β-Estradiol (E2) and 4-nitrophenyl phosphate were purchased from Sigma-Aldrich (Schnelldorf, Germany), while the lipopolysaccharide (LPS; from *E. coli*) and dexamethasone (Dexa) were acquired from Sigma-Aldrich (Steinheim, Germany). Normocin, zeocin and Quanti-Luc™ were from Invivogen (Toulouse, France). Triton®X 100 and dimethyl sulfoxide (DMSO) were purchased from Carl Roth GmbH&Co (Karlsruhe, Germany). Reference standards of *Alternaria* toxins were obtained from several suppliers or kindly provided by other researchers. Interested readers may refer to Puntscher et al. (2019a) for details. The mycotoxins AOH, ATX-I, ALTP and TeA, tested for their immunomodulatory and/or antiestrogenic effects, were acquired from Sigma-Aldrich (Schnelldorf, Germany), Szabo-Scandic (Vienna, Austria), Cfm Oskar Tropitzsch (Marktredwitz, Germany), and Santa Cruz Biotechnology (Heidelberg, Germany), respectively. For LC–MS/MS analyses, acetonitrile (ACN) and water (both LC–MS grade) were purchased from Honeywell (Seelze, Germany). For the SFC-based fractionation of CE, compressed CO_2_ (4.5 grade, purity ≥ 99.995%) was purchased from Messer (Frankfurt am Main, Germany) and methanol (ultrahigh-gradient grade) was purchased from Merck (Darmstadt, Germany).

### Complex extract of *Alternaria* mycotoxins and AOH-spiked *Alternaria* extract

The CE employed in the present study was previously obtained by growing the *Alternaria alternata* strain DSM 62010 on long rice for 21 days, and chemically characterized by LC–MS/MS analysis (Puntscher et al. 2019a, b). Concentrations of the mycotoxins in CE at the highest concentration applied in the NF-κB assay (20 µg/mL) are reported in Table 1. To assess the contribution of AOH to the immunosuppressive properties of CE (characterized by a low concentration of the mycotoxin), an AOH-spiked *Alternaria* extract (SE) was prepared by dissolving pure AOH in CE, such that 20 µg/mL of CE contained 20 µM AOH, 10 µg/mL of CE contained 10 µM AOH, and so forth. Prior to the experiments, the SE was analyzed by LC–MS/MS to ensure the correct addition of AOH to the extract.

**Table 1:** Concentration of *Alternaria* mycotoxins to which THP1-Lucia™ cells were exposed during treatment with the highest CE concentration tested (20 µg/mL)

Mycotoxins	Concentration (nM)
Alternariol (AOH) ^a^	60
Alternariol monomethyl ether (AME)	48
Altenuene (ALT)	52
Tenuazonic acid (TeA)	60,600
Tentoxin (TEN)	0.8
Altertoxin-I (ATX-I)	564
Altertoxin-II (ATX-II)	804
Alterperylenol (ALTP)	720
Stemphyltoxin-III (STTX-III)	1200
Altenusin (ALS)	20
Altersetin (AST)	920

^a^Contrary to CE, the concentration of AOH in treatments with 20 µg/mL SE was 20 µM

### Fractionation of the *Alternaria* extract by supercritical fluid chromatography

For the fractionation of the CE, a semi-preparative SFC was used (Prep-15 SFC system, Waters, Milford, MA, USA). The system comprises a fluid delivery module connected to an Accel 500 LC chiller, a Waters 2767 sample manager, a ten-port column oven, a back pressure regulator, a heat exchanger, a make-up pump, a Waters 2998 PDA, and a Waters 2424 ELSD. The system was operated by the software MassLynx V4.1 and FractionLynx. A Torus 1-Aminoanthracene column (5 μm, 10 mm × 250 mm; Waters Milford, MA, USA) was used at a column temperature of 40 °C with pure CO_2_ as solvent A and methanol as solvent B applying the following gradient: 0–4.0 min from 5 to 50% B; 4.0–5.0 min 50% B; 5.0–6.0 min ramp down to 5% B, and 6.0–7.0 min 5% B as equilibration step. For fractionation CE was diluted with a solvent composed of 70% hexane and 30% isopropanol (*v/v*) prior to injection (100 µL). The flow rate was 15 mL/min, the backpressure was set to 120 bar and after the column the eluents were combined with a methanol make-up flow of 5 mL/min to facilitate the detection and fraction collection. The retention time stability was checked each time prior fraction collection. A representative chromatogram, recorded at a wavelength of 205 nm, indicating the sampling events is presented in Supplementary Figure S1. Separation performance was monitored with an ultra-high-performance supercritical fluid chromatograph (UHPSFC, Acquity UPC2-DAD/ELSD/MS, Waters, Milford, MA, USA). The eleven collected fractions (F1-F11) were dried under nitrogen flow at room temperature, followed by resuspension in DMSO to obtain stock solutions with a concentration of 3 mg/mL.

### LC–MS/MS analysis for the quantification of mycotoxins contained in the CE-fractions

For the quantification of mycotoxins in the eleven collected CE-fractions, LC–MS/MS analyses were performed using the method developed by Puntscher et al (2018). This method has been validated for the analysis of mycotoxins in food matrices and further applied for the analysis of urine, feces (Puntscher et al. 2019a, b), and cell culture media (Crudo et al. 2020). Briefly, CE-fractions were diluted with a solvent consisting of 50% acetonitrile and 50% water (*v/v*) and analyzed by using a high-performance liquid chromatographic system (HPLC, UltiMate3000, Dionex Thermo Fisher Scientific, Vienna, Austria) coupled to a TSQ Vantage triple quadrupole mass spectrometer equipped with a heated electrospray ionization (HESI) interface (Thermo Fisher Scientific). For the chromatographic separation, a Supelco Ascentis Express column (C18, 2.7 μm, 10 cm × 2.1 mm) equipped with a Phenomenex SecurityGuard™ precolumn (C18, 2 mm, Phenomenex, Torrance, CA) was used. Data were acquired in negative electrospray ionization mode. Further details are reported in Puntscher et al. (2018).

To avoid carry-over phenomena and verify the overall performance of the instrument, solvent blanks were routinely injected. Samples were randomly analyzed, and quantification of the analytes was performed by external calibration (calibration set injected after every approximately 20 samples). For instrument control, data acquisition and data evaluation, Chromeleon™ chromatography data system software (v. 6.80 SR13 Build 3818), Xcalibur™ software (v. 3.0, Thermo Scientific), and TraceFinder™ software (v. 3.3, Thermo Scientific) were used, respectively.

### NF-κB gene reporter assay

THP1-Lucia™ NF-κB monocytes, obtained by transfection of THP-1 cells with an NF-κB–inducible luciferase reporter construct, were purchased from InvivoGen (Toulouse, France) and cultivated following the provider’s instructions. Briefly, cells were routinely maintained in Roswell Park Memorial Institute (RPMI)-1640 medium containing 25 mM HEPES buffer, 10% (*v/v*) heat-inactivated fetal bovine serum (FBS), 1% penicillin/streptomycin solution (100 U/mL) and 100 µg/mL normocin. Cells were sub-cultivated twice a week and, to maintain selection pressure, 100 µg/mL zeocin were added to the flask every other passage.

For the experiments, 1 × 10^5^ cells/well were seeded into 96-well plates and simultaneously exposed to the extracts (CE and SE; 0.0001–20 µg/mL), fractions of CE (F1–F11; 0.075–7.5 µg/mL), single mycotoxins (TeA, 0.1–250 µM; AOH, 0.0001–20 µM; ATXI and ALTP, 0.001–20 µM), solvent control (0.25% DMSO), or negative control (1 µM Dexa). For the positive and negative control treatments, 10 ng/mL LPS from *E. coli* were added to the cells 2 h after the start of the incubation. To assess the ability of the test compounds/extracts/fractions to activate the NF-κB pathway, cells were incubated for 20 h at 37 °C and 5% CO_2_ without the addition of LPS. On the contrary, their immunosuppressive properties were tested by adding 10 ng/mL LPS (after 2 h, as for the positive control) and incubating the cells for a further 18 h. The DMSO concentration in the various treatments was always 0.25%. At the end of the incubation time (20 h), the luciferase activity was assessed by employing the coelenterazine-based luminescence reagent “Quanti-Luc™”. Briefly, the plate was centrifuged at 140 rcf for 2 min, after which 10 µL of the cell supernatants were transferred to a white 96-well plate. The luminescence measurement was performed, after the automatic addition of 50 µL/well of the detection reagent, with a microplate reader equipped with an injection system (BioTec Synergy H1; Agilent Technologies, Santa Clara, USA). Every condition was tested in three–six technical replicates and in at least three independent experiments.

### Alkaline phosphatase assay

To assess the estrogenic and antiestrogenic properties of the CE-fractions (0.015–1.5 µg/mL) and single mycotoxins (ATX-I and ALTP; 0.0002–10 µM), the human endometrial adenocarcinoma Ishikawa cell line was employed (European Collection of Authenticated Cell Cultures; Wiltshire, United Kingdom). This estrogen-sensitive cell line was chosen as a model system because of the constitutive expression of both ERα and ERβ (Boehme et al. 2009). Cells were routinely maintained at 37 °C and 5% CO_2_ in Minimum Essential Medium containing phenol red and supplemented with 5% FBS (*v/v*), 1% penicillin/streptomycin (*v/v*; 100 U/mL) and 1% L-glutamine (*v/v*). Cells were sub-cultured every third to fourth day at a confluency of approximately 80%.

For the AlP assay, cells in assay medium (DMEM/F-12 without phenol red supplemented with 5% charcoal/dextran–treated FBS) were seeded in 96-well plates at a density of 1 × 10^4^ cells/well, followed by incubation at 37 °C and 5% CO_2_ for 48 h prior to cell treatment. As positive and solvent controls, 1 nM E2 and 0.1% DMSO were used, respectively. To assess the antiestrogenic properties of the various test conditions, cells were co-incubated with 1 nM E2, while no E2 was added for the assessment of the estrogenic properties. All conditions were tested in at least three technical replicates and in three independent experiments. After 48 h exposure of cells to the various test conditions (with and without co-incubation with 1 nM E2), the cells were washed three times with phosphate-buffered saline (PBS) and lysed by shock-freezing at −80 °C for 20 min. Cells were thawed at room temperature for 5 min, followed by the addition of 50 µL/well of an AlP buffer containing 5 mM 4-nitrophenylphosphate, 1 M diethanolamine, and 0.24 mM MgCl_2_ (pH 9.8). After 5 min of incubation at room temperature, the AlP activity was determined using a microplate reader (BioTec Synergy H1; Agilent Technologies, Santa Clara, USA) by measuring the absorbance at 405 nm every 2 min for 1 h. The slope of the curves within the linear range was calculated as a measure of the enzyme’s activity.

### Celltiter-blue® (CTB) cell viability assay

To assess the impact of test compounds/extracts/fractions on the viability of THP1-Lucia™ and Ishikawa cells, the CTB cell viability assay was performed. For THP1-Lucia™ cells, two hours before the end of the incubation time, a positive control for cytotoxicity was prepared by exposing untreated cells to 0.01% Triton X. At the end of the incubation period, the CTB reagent was added to each well of the plate at a 1:10 dilution, and the cells were then incubated at 37 °C and 5% CO_2_ for an additional 2 h. Plates were centrifuged at 140 rcf for 2 min, followed by transferring 100 µL of cell supernatants to a black 96-well plate. Ishikawa cells were exposed to the various test conditions and controls for 48 h. Afterwards test media were replaced with 100 µL/well of CTB solution (CTB reagent diluted 1:10 with phenol red-free DMEM/F12 medium). As a positive control served 0.005% Triton-X ®. After 50 min incubation at 37 °C and 5% CO_2_, 100 µL of cell supernatants were transferred to a black 96-well plate.

For both cell lines, the fluorescence intensity was measured at an excitation wavelength of 530 nm and an emission wavelength of 560 nm using a microplate reader. Every condition in each experiment was tested in three technical replicates, and the data were reported as the mean value (+ standard deviation) of at least three independent experiments.

### Statistical analysis

Independent Student *t*-test was performed to determine significant differences between the treatments and the respective positive or solvent controls. To assess significant differences among the various concentrations of each tested mycotoxin, extract, or fraction, analysis of variance (one-way ANOVA) with Fisher-LSD post hoc test was performed. Samples were considered significantly different for *p < 0.05, **p < 0.01, or ***p < 0.001. All statistical tests were conducted using OriginPro 2021 software (v. 9.8.0.200, OriginLab Corporation, Northampton, MA, USA).

## Results

### Immunomodulatory effects of AOH, CE and SE

**Fig. 1:**
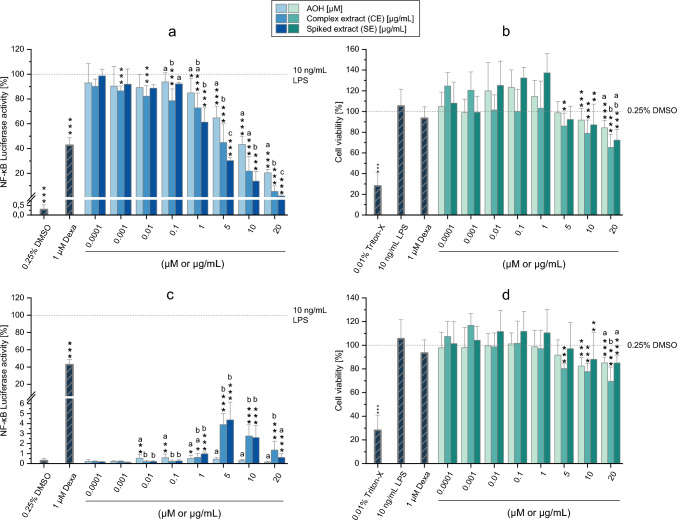
Immunomodulatory and cytotoxic properties of AOH, CE and SE in THP1-Lucia™ monocytes in the presence and absence of LPS stimulation **a** and **b** show the results of the NF-κB and CTB assays (respectively) in the presence of LPS stimulation, while **c** and **d** refer to the treatments in the absence of LPS stimulation. Results are expressed as mean + SD of at least 3 biological replicates and are normalized to the positive control (10 ng/mL LPS; in the NF-κB assay) or solvent control (0.25% DMSO; in the CTB assay). Dexamethasone (1 µM Dexa; for the NF-κB assay) and Triton-X (0.01%; for the CTB assay) were used as controls. Statistically significant differences compared to the positive control (**a, c**) or solvent control (**b, d**) were assessed by applying the Student *t*-test (*p < 0.05; **p < 0.01; ***p < 0.001). In **b** and **d** bars are marked with stars only in case of a significant decrease in cell viability. Significant differences between AOH, CE and SE at each concentration were assessed by applying the one-way ANOVA with Fisher-LSD post hoc test (**a-c**; *p* < 0.05). Bars are marked with letters only in case of a significant difference between the groups. Different letters above the bars indicate statistically significant difference at *p* < 0.05

To assess the immunosuppressive and immunostimulatory effects of CE, AOH and SE, the NF-κB reporter gene assay in THP1-Lucia™ monocytes was performed in the presence and absence of LPS stimulation, respectively. With respect to CE, concentrations ranging from 0.0001 to 20 µg/mL were tested. As shown in Fig. 1a, the results of the assay clearly showed the ability of CE to suppress the LPS-induced activation of the NF-κB pathway starting from a concentration of 0.001 µg/mL (*p* < 0.01). However, a substantial and concentration-dependent decrease in pathway activation was only observed in the range of 1–20 µg/mL. To exclude a possible misinterpretation of the results due to cytotoxicity, the CTB assay was carried out in parallel. Results of the CTB assay (Fig. 1b) showed a significant decrease in cell viability only after exposure of cells to the three highest concentrations tested (5 µg/mL, viability: 85.9 ± 12.4%; 10 µg/mL, 78.9 ± 12.9%; 20 µg/mL, 65.3 ± 12.6%). To assess the contribution of the *Alternaria* mycotoxin AOH to the immunosuppressive effects of CE, concentrations of SE in the range 0.0001–20 µg/mL (AOH-spiked extract containing 0.0001–20 µM AOH) and the corresponding concentrations of AOH (0.0001 to 20 µM) were also tested (Fig. 1a). The exposure of cells to 1 µM (p < 0.05) and 5–20 µM (*p* < 0.001) AOH resulted in a concentration-dependent suppression of the NF-κB pathway, measured as an attenuated luciferase activity. The reduction in luciferase activity was higher than that of the negative control (1 µM Dexa) only at the highest concentration tested (20 µM; around 80% of reduction). Concerning the cytotoxic effects, a slight but significant decrease in cell viability was observed at the two highest concentrations tested (Fig. 1b; cell viability: 84.3 ± 7.1% at 20 µM). Exposure of cells to SE resulted in a concentration-dependent suppression of the LPS-induced activation of the NF-κB pathway in the concentration range 1–20 µg/mL (Fig. 1a). Of note, the suppressive effects of SE were stronger compared to those induced by AOH alone, but similar to the effects caused by CE (which contains a minimal quantity of AOH). Except for the two highest concentrations of SE, which moderately suppressed cell viability (10 µg/mL: viability 87 ± 19%; 20 µg/mL: viability: 72 ± 10%), no other significant changes in viability were observed compared to the solvent control.

With respect to treatments without LPS-stimulation, results reported in Fig. 1c show the inability of AOH, CE and SE to substantially activate the NF-κB pathway. In fact, despite significant increases in luciferase activity were observed at specific concentrations of AOH, CE and SE (compared to the solvent control), these increases never reached values above 4.4 ± 1.8% (recorded for SE at 5 µg/mL). Similar to the results obtained in the presence of LPS stimulation, reductions in cell viability were only detected at concentrations ≥ 5 µM for CE, and ≥ 10 µM for AOH and SE (Fig. 1d). Of note, viabilities below 80% were only observed at the two highest concentrations of the CE.

### Quantification by LC–MS/MS of mycotoxins in the CE-fractions

The CE-fractions obtained by SFC and resuspended in DMSO at a concentration of 3 mg/mL were analyzed by LC–MS/MS analysis to obtain quantitative information about their mycotoxin profiles. As reported in Table 2, which shows the concentration (in µM) of *Alternaria* mycotoxins in the stock solutions of the CE-fractions (*c* = 3 mg/mL), ten out of the eleven *Alternaria* mycotoxins originally contained in the CE (see Table 1) were detected and quantified. Of note, the epoxide-carrying perylene quinone STTX-III was not found in any of the fractions, while the *Alternaria* toxin altenuic acid III (AA-III) was detected for the first time in the CE-fractions F8 and F9. To facilitate the comparison and interpretation of the NF-κB and AlP assay results in relation to the actual concentrations of mycotoxins to which the cells were exposed during treatment with the CE-fractions, the specific mycotoxin concentrations encountered by the cells are given in Supplementary Tables S1 and S2, respectively.

**Table 2:** Concentration of *Alternaria* mycotoxins in the stock solutions (*c* = 3 mg/mL) of the CE-fractions

Fraction	Mycotoxin concentration (µM) ^a^										
	AOH	AME	ALT	TeA	TEN	ATX-I	ATX-II	ALTP	ALS	AA-III	AST
F1				27.3		1.1		11			
F2				8110							
F3		0.1		3610	0.1			0.9			
F4		0.1		3090	0.8			0.6			150
F5		0.1		1880	2.3	0.4		0.7			100
F6		0.1		2510	0.3	0.3		0.9			72
F7		0.1	219	2530	0.1	0.4	0.9	0.8	3.6		21
F8	1.1	22		2150		0.7		3.4		11	3.8
F9	44	2.4	3.3	1610		1.3	110	5.2	15	13	5.7
F10	6.0	0.8		1100		810	53	410	150		2.8
F11	1.9	0.6	< 0.1	1290		40	15	250	3.4		2.9

^**a**^The reported concentrations are 400 and 2000 times higher than the concentrations tested in the AlP and NF-κB assays, respectively. Values < LOQ are not displayed

### Immunomodulatory effects of CE-fractions

**Fig. 2:**
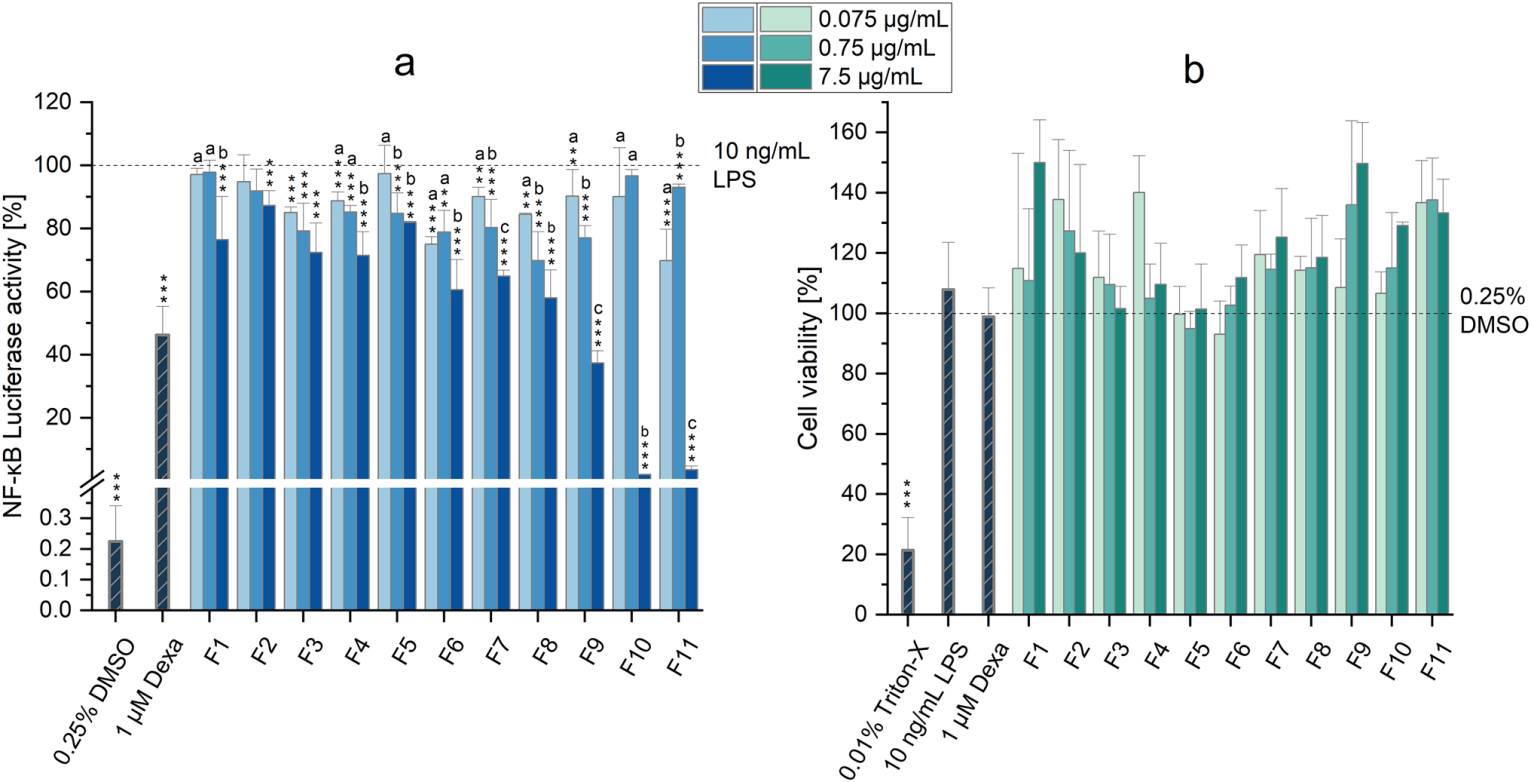
Effects of CE-fractions on the LPS-induced NF-κB pathway activation and viability of THP1-Lucia™ monocytes. **a** and **b** show the results of the NF-κB and CTB assays, respectively. Results are expressed as mean + SD of at least 3 biological replicates and are normalized to the positive control (10 ng/mL LPS; in the NF-κB assay) or solvent control (0.25% DMSO; in the CTB assay). Dexamethasone (1 µM Dexa; for the NF-κB assay) and Triton-X (0.01%; for the CTB assay) were used as controls. Statistically significant differences compared to the positive control (in the NF-κB assay) or the solvent control (in the CTB assay) were assessed by applying the Student *t*-test (*p < 0.05; **p < 0.01; ***p < 0.001). Significant differences among the various concentrations of the fractions tested were assessed by applying the one-way ANOVA with Fisher-LSD post hoc test. Bars are marked with letters only in case of a significant difference between the groups. Different letters above the bars indicate statistically significant differences at *p* < 0.05

To identify the *Alternaria* mycotoxins involved in the suppression by CE of the LPS-induced activation of the NF-κB pathway, the eleven CE-fractions (F1-F11) obtained by SFC were individually tested in the NF-κB gene reporter assay. Figure 2a shows the results of the NF-κB reporter gene assay obtained by exposing LPS-stimulated THP1-Lucia™ monocytes to three different concentrations of the CE-fractions (i.e. 0.075, 0.75, and 7.5 µg/mL), while the corresponding CTB results are reported in Fig. 2b.

The results obtained clearly demonstrate the ability of the majority of CE-fractions to suppress the LPS-induced NF-κB pathway activation, albeit with varying potencies. Except for the fractions F2 and F3, for which no significant differences among the concentrations tested were observed, treatment of cells with the highest concentration of the various fractions (7.5 µg/mL) resulted in stronger immunosuppressive effects compared to the lowest tested concentrations. Of note, the most relevant suppressive effects were observed in cells exposed to 7.5 µg/mL of the fractions F9, F10 and F11, for which a luciferase activity of 37.6 ± 3.9%, 2.0 ±  < 0.1%, and 3.5 ± 1.2% was observed, respectively. To prevent misinterpretation of the results due to cytotoxicity, the CTB assay was consistently conducted in parallel. As shown in Fig. 2b, none of the fractions or concentrations tested exhibited cytotoxic effects, thereby ruling out cytotoxicity as a potential explanation for the observed suppression of NF-κB pathway activation.

Despite the poor indication of a possible presence in the CE of immunostimulatory compounds (Fig. 1c), the various CE-fractions were also tested for their immunostimulatory effects. Results obtained revealed the ability of the fractions F1, F7–F11 to significantly increase the luciferase activity (Supplementary Figure S2a). However, the only fraction able to induce an increase in luciferase activity above 5% was the fraction F9, for which a luciferase activity of 26.6 ± 5.2% was detected at the highest concentration tested (7.5 µg/mL; Supplementary Figure S2a). Of note, no cytotoxic effects were observed after exposure of cells to the different CE-fractions, except for the fractions F5 and F6 for which slight reductions in cell viability were observed (Figure S2b; viability > 80%).

### Immunomodulatory effects of ATX-I, ALTP and TeA

**Fig. 3:**
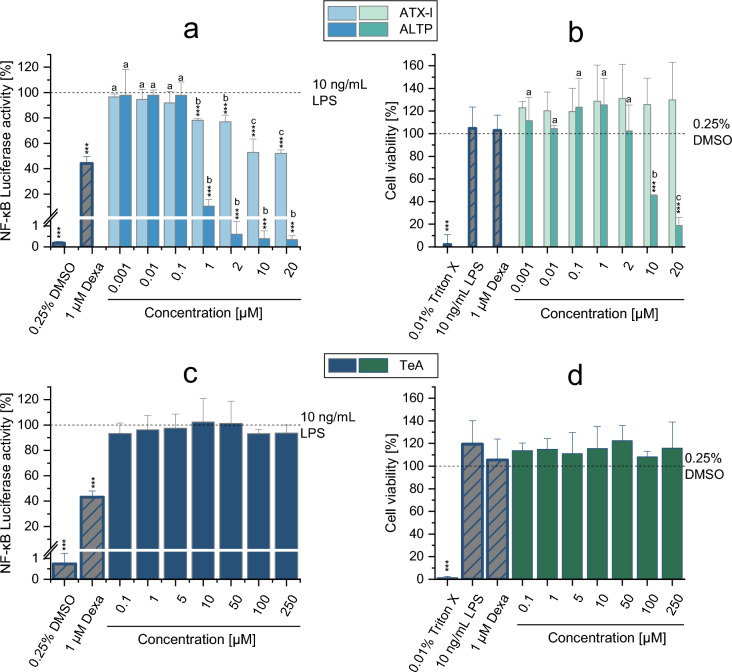
Effects of ATX-I, ALTP, and TeA on the LPS-induced NF-κB pathway activation and viability of THP1-Lucia™ monocytes. **a** and **b** show the results of the NF-κB and CTB assays (respectively) obtained from the exposure of cells to ALTP and ATX-I, while c) and d) report the results for TeA. Results are expressed as mean + SD of at least 3 biological replicates and are normalized to the positive control (10 ng/mL LPS; in the NF-κB assay) or solvent control (0.25% DMSO; in the CTB assay). Dexamethasone (1 µM Dexa; for the NF-κB assay) and Triton-X (0.01%; for the CTB assay) were used as controls. Statistically significant differences compared to the positive control (in the NF-κB assay) or the solvent control (in the CTB assay) were assessed by applying the Student *t*-test (*p < 0.05; **p < 0.01; ***p < 0.001). Significant differences among the various concentrations of each mycotoxin were assessed by applying the one-way ANOVA with the Fisher-LSD post hoc test. Bars are marked with letters only in case of significant differences among the concentrations tested. Different letters above the bars indicate statistically significant difference at *p* < 0.05

Based on the results of the NF-κB gene reporter assay obtained by exposing THP1-Lucia™ monocytes to the various CE-fractions, and considering the data of mycotoxin concentrations acquired by LC–MS/MS analysis (Table 2 and Supplementary Table S1), the *Alternaria* mycotoxins ALTP and ATX-I (0.001 to 20 µM), as well as TeA (0.1 to 250 µM), were selected for further investigations of their immunomodulatory properties. Results of the NF-κB assay and CTB assay in the presence of LPS stimulation are reported in Fig. 3. As shown in Fig. 3a, exposure of cells to the perylene quinones ALTP and ATX-I resulted in a concentration-dependent suppression of the LPS-induced NF-κB pathway activation at concentrations ≥ 1 µM (*p* < 0.001). In particular, while exposure of cells to ATX-I led to suppressions of 52.8 ± 10.6% and 52.0 ± 1.9% at the two highest concentrations tested (10 and 20 µM, respectively), a suppression of about 90% of the luciferase activity was observed already after treatment of THP1-Lucia™ cells with 1 µM ALTP. Of note, the treatment of cells with 2–20 µM ALTP resulted in a complete suppression of the pathway, since no significant differences compared to the solvent control were observed. It has to be pointed out that while exposure of cells to the various ATX-I concentrations did not show any cytotoxic effects, exposure to 10 µM and 20 µM ALTP resulted in strong cytotoxicity (cell viability: 45.2 ± 0.9% and 18.9 ± 7.1%, respectively) (Fig. 3b). Contrary to what was observed for the perylene quinones, the *Alternaria* mycotoxin TeA did not suppress the NF-κB pathway even at the highest concentration tested (250 µM; Fig. 3c). No reduction in cell viability was observed following exposure of cells to the various concentrations of TeA (Fig. 3d).

The *Alternaria* mycotoxins ATX-I, ALTP, and TeA were also tested in the absence of LPS stimulation to assess their possible immunostimulatory properties. As shown in Supplementary Figure S3, none of these mycotoxins triggered activation of the NF-κB pathway. Furthermore, their effects on cell viability were similar to those observed with LPS stimulation.

### Estrogenic and antiestrogenic properties of CE-fractions

**Fig. 4:**
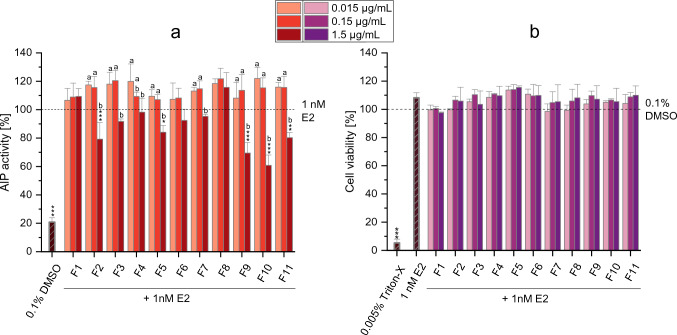
Antiestrogenic properties of CE-fractions in Ishikawa cells. **a** and **b** show the results of the AlP assay and CTB assay of the SFC fractions (0.015; 0.15; 1.5 µg/mL), respectively. Results are expressed as mean + SD of at least 3 biological replicates and data were normalized to the positive control (1 nM E2; in the AlP assay) or solvent control (0.1% DMSO; in the CTB assay). Triton-X (0.005%) served as a positive control in the CTB assay. Statistically significant differences compared to the positive control (in the AlP assay) or the solvent control (in the CTB assay) were assessed by applying the Student *t*-test. Bars are marked with stars only in case of a significant decrease compared to the control (*p < 0.05; **p < 0.01; ***p < 0.001). Significant differences among the various concentrations of each fraction were evaluated by applying the one-way ANOVA and the Fisher-LSD post-hock test. Bars are marked with letters (**a–b**) only in case of significant differences among the concentrations tested. Different letters above the bars indicate statistically significant difference at *p* < 0.05

Besides examining their immunomodulatory properties, the CE fractions were also employed to investigate potential interference with the estrogenic pathways. For this purpose, the AlP assay was conducted using Ishikawa cells exposed to 1.5, 0.15, and 0.015 µg/mL of the fractions. Incubations were carried out with and without simultaneous co-incubation with 1 nM E2 to assess their anti-estrogenic and estrogenic effects, respectively. As shown in Fig. 4a, five out of eleven fractions (i.e. F2, F5 and F9-11) were found to significantly suppress the alkaline phosphatase activity in cells co-exposed to 1 nM E2. Of note, the anti-estrogenic effects of these fractions were only observed at the highest concentration applied (1.5 µg/mL), with the fractions F9 and F10 showing the strongest suppressive effects (AlP activity: 69.5 ± 7.4% and 60.8 ± 7.3%, respectively). As reported in Fig. 4b, none of the tested fractions exerted cytotoxic effects. With respect to the estrogenic properties of the CE fractions, none of the fractions and concentrations resulted in a significant increase in AlP activity compared to the DMSO control (Supplementary Figure S4a). Similarly to what was observed during co-treatment with E2, no cytotoxic effects were observed (Supplementary Figure S4b).

### Estrogenic and antiestrogenic properties of ATX-I and ALTP

**Fig. 5:**
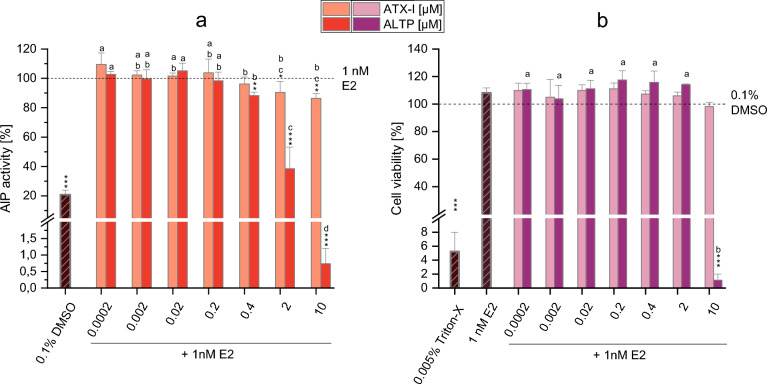
Antiestrogenic properties of ATX-I and ALTP in Ishikawa cells. **a** and **b** show the results of the AlP assay and CTB assay (respectively) during co-incubation of cells with the mycotoxins and 1 nM E2. Results are expressed as mean + SD of at least 3 biological replicates and data were normalized to the positive control (1 nM E2; in the AlP assay) or solvent control (0.1% DMSO; in the CTB assay). Triton-X (0.005%) served as a positive control in the CTB assay. Statistically significant differences compared to the positive control (in the AlP assay) or the solvent control (in the CTB assay) were assessed by applying the Student *t*-test. Bars are marked with stars only in case of a significant decrease compared to the control (*p < 0.05; **p < 0.01; ***p < 0.001). Significant differences among the various concentrations of each mycotoxin were evaluated by applying the one-way ANOVA and the Fisher-LSD post hock test. Bars are marked with letters (**a–b**) only in case of significant differences among the concentrations tested. Different letters above the bars indicate statistically significant difference at *p* < 0.05

Based on the results of the AlP assay of the various CE-fractions, and considering the data of mycotoxin concentrations acquired by LC–MS/MS analysis (Table 2 and Supplementary Table S2), the *Alternaria* mycotoxins ALTP and ATX-I (0.0002 to 10 µM) were tested for their possible antiestrogenic effects. As shown in Fig. 5a, exposure of cells to ATX-I resulted in a slight but significant reduction of the AlP activity (compared to the positive control; 1 nM E2) only at the two highest concentrations tested (i.e. 2 and 10 µM). The AlP activity was indeed significantly reduced to 90.4 ± 7.4% (*p* < 0.05) at 2 µM and to 86.4 ± 3.3% (*p* < 0.01) at 10 µM of ATX-I. Of note, none of the tested concentrations of ATX-I affected cell viability (Fig. 5b), thus excluding the possibility of misinterpreting the AlP results due to cytotoxicity. With respect to the perylene quinone ALTP, exposure of cells to 0.4, 2, and 10 µM of the mycotoxin resulted in a significant and concentration-dependent decrease in AlP activity (AlP activity: 88.2 ± 2.2%, *p* < 0.01; 38.5 ± 14.6%, *p* < 0.001; 0.74 ± 0.45%,* p* < 001; respectively) compared to the treatment with 1 nM E2 alone (Fig. 5a). However, the results of the CTB assay showed the induction of strong cytotoxic effects by the highest ALTP concentration tested (10 µM; Fig. 5b).

## Discussion

Molds of the genus *Alternaria* produce mixtures of mycotoxins that have been reported to exert a wide variety of toxic effects, including genotoxicity, mutagenicity, and endocrine disruption (Schrader et al. 2001; Brugger et al. 2006; Tiemann et al. 2009; Louro et al. 2024). Despite the possible deleterious effects on human health, the immunotoxic properties of this class of contaminants have rarely been investigated. In this context, an important pathway that plays a significant role in regulating immune responses is the NF-κB pathway, whose activation regulates the expression of genes involved in immune responses, inflammation, cell survival, and proliferation (Biswas and Bagchi 2016). In the present study, a complex *Alternaria* mycotoxin extract (CE) containing, among others, eleven chemically characterized mycotoxins, was employed to assess the ability of this class of mycotoxins to target the NF-κB pathway, with the final aim of identifying mycotoxins with immunotoxic properties. As shown in Fig. 1a, the CE was found to suppress the LPS-induced activation of the NF-κB pathway in THP1-Lucia™ monocytes in a concentration-dependent manner. In particular, suppressive effects were observed in the concentration range 0.001–20 µg/mL, and treatments with the highest concentration tested resulted in an almost complete suppression of the pathway. Despite the slightly reduced cell viability might have contributed to the suppression in the NF-κB pathway observed at the three highest CE concentrations tested (5 µg/mL, 10 µg/mL, 20 µg/mL; Fig. 1b), no decrease in cell viability was observed at the lowest active concentrations, confirming the presence of compounds in the CE able to directly target the NF-κB pathway. Differently from the treatments with LPS-stimulation, which showed significant suppressive effects, only slight and negligible increases in luciferase activity were detected in the absence of LPS-stimulation (< 5%; Fig. 2c). Thus, the data obtained point toward the presence of known or unknown *Alternaria* mycotoxins in the CE that predominantly exhibit immunosuppressive properties.

In this context, among the various *Alternaria* mycotoxins contained in the CE (Table 1), the mycotoxin AOH was previously reported to suppress the activation of the NF-κB pathway in THP-1-derived macrophages starting from a concentration of 1 µM (Kollarova et al. 2018), as well as to reduce the LPS-induced secretion of tumor necrosis factor-α (TNF-α) in THP-1 monocytes (Solhaug et al. 2016). In the present study, the effects of 0.0001–20 µM AOH on non-differentiated THP1-Lucia™ monocytes were therefore assessed to examine its contribution to the immunosuppressive effects of CE. Consistent with the results from Kollarova et al. (2018), we found AOH to suppress the NF-κB pathway in monocytes starting from a concentration of 1 µM, with the strongest suppression observed at the highest concentration tested (20 µM; ≈80% reduction; Fig. 1a). It must be pointed out that, despite the significant (although negligible) decrease in cell viability observed at the two highest concentrations tested, which might have partially contributed to the suppressive effects on the NF-κB pathway induced by the mycotoxin, no reduction in viability was observed at the lowest active concentrations, confirming the ability of the toxin to target the pathway. Considering the LC–MS/MS results regarding the concentration of AOH in the CE (Table 1), cells treated with 5 µg/mL of the CE, which significantly reduced NF-κB pathway activation, were exposed to an AOH concentration of 0.015 µM. Since this concentration is much lower than the 1 µM needed to induce immunosuppressive effects (Fig. 1a), it is likely that other compounds in the CE either possess immunosuppressive properties or enhance the effects of AOH within the complex mixture. To better assess the contribution of AOH to the immunosuppressive effects of the CE, an AOH-spiked extract (SE) was prepared such that it contained an AOH concentration of 20 µM in treatments with 20 µg/mL SE, 10 µM in treatments with 10 µg/mL SE, and similarly for the other concentrations tested. As shown in Fig. 1a, exposure of monocytes to 0.0001–20 µg/mL SE resulted in a concentration-dependent suppression of the LPS-induced NF-κB pathway activation starting from 1 µg/mL. These suppressive effects can partially be traced back to a reduced cell viability only in the case of treatments with 10 µg/mL and 20 µg/mL SE since no reduction in cell viability was observed at the lowest active concentrations. Comparing the results obtained for AOH, CE and SE, significant differences in the immunoinhibitory effects were observed in most cases. In particular, SE-induced suppressive effects were always found to be significantly stronger than those exerted by the respective concentrations of AOH. As an example, while treatment with 5 µg/mL SE (containing 5 µM AOH) resulted in a luciferase activity of 30.27 ± 2.70%, exposure of cells to 5 µM AOH only reduced the bioluminescence signal to 64.75 ± 9.36%. These findings confirm the known immunosuppressive properties of AOH and the presence of further metabolites in the extract able to suppress the NF-κB pathway. It should be noted that, unlike previous observations with SE and AOH treatments, significant differences in luciferase signal suppression between CE and SE treatments were not always found and, when present, were less pronounced than those observed between SE and AOH. Based on this and considering the low content of AOH in the CE (Table 1), the contribution of AOH to the immunosuppressive effects of the CE might therefore be very limited.

To identify the compounds involved in the immunosuppressive effects of the extract, fractions of the CE obtained by SFC were tested in the NF-κB assay. It should be noted that, after the fractionation procedure, the epoxide-carrying perylene quinone STTX-III was not found in any of the fractions, while the *Alternaria* toxin AA-III was detected for the first time in CE-fractions F8 and F9. The detection of AA-III in these fractions was likely due to the fractionation process, which concentrated the mycotoxin to a level above its detection limit. In contrast, the absence of STTX-III in the collected CE fractions can likely be attributed to its high reactivity due to the presence of an epoxy group in its chemical structure. Indeed, STTX-III has previously been reported to be highly unstable, even in solvents and buffers (Zwickel et al. 2016; Crudo et al. 2020). Interestingly, despite containing an epoxy group, the mycotoxin ATX-II remained present after fractionation. This difference in stability was also observed in a previous study from our group (Crudo et al. 2020), where a reduction in STTX-III content, but not in ATX-II, was observed after 3 h of incubation of the CE with PBS.

Regarding the impact of CE fractions on the NF-κB activity, most fractions resulted in suppression of LPS-induced activation of the NF-κB pathway, with fractions F9, F10, and F11 being the most effective (Fig. 2). According to the mycotoxin quantification performed by LC–MS/MS (Table 2), the mycotoxins ALS, ALTP, AOH, ATX-I, ATX-II, AA-III and TeA were among the most abundant in those fractions. It should be noted that the minimal role of AOH in the overall immunosuppressive effects of the CE was also confirmed through the analysis of the CE fractions. Indeed, the active fraction F9, characterized by the highest level of AOH among all fractions, only contained 0.1 µM of the mycotoxin (Table S1), a concentration that falls far below the range known to inhibit the NF-κB pathway (Fig. 1a). As shown in Figure S5, apart for AOH, fraction F9 also contained ~ 62% of the total ATX-II content found in the CE-fractions, as well as 55% of AA-III, suggesting that these mycotoxins could possibly be involved in the effects observed. Despite AA-III and ATX-II could not be tested in the present study due to their lack of commercial availability, ATX-II was previously reported to suppress the NF-κB pathway in THP1-Lucia™ monocytes in the absence of LPS stimulation (Del Favero et al. 2020). However, this suppression failed in the concentration range of 0.1–1 µM during co-incubation with 100 µg/mL LPS (Del Favero et al. 2020). While this might suggest ATX-II’s inability to counteract the effects induced by LPS, it is important to note that Del Favero et al. (2020) conducted their experiments using a concentration of LPS ten times higher than that utilized in the present study (10 ng/mL). Based on this, possible immunosuppressive effects by the mycotoxin in the presence of lower LPS concentrations cannot be completely excluded. Further studies are therefore required to better assess the potential role of ATX-II, as well as AA-III, in the immunosuppressive effects of fraction F9. Apart for the fraction F9, treatment of cells with the fractions F10 and F11 caused an almost complete suppression of the NF-κB pathway at the highest concentration tested (7.5 µg/mL). Among the various *Alternaria* mycotoxins, fractions F10 and F11 were found to comprise significant amounts of ALTP (1 µM and 0.6 µM, respectively), while fraction F10 also contained the highest amount of ATX-I (2 µM) among the various CE-fractions. Building upon these observations, ALTP and ATXI were chosen to be tested in the NF-κB assay individually. Furthermore, despite the low likelihood of functioning as an immunosuppressive compound due to its presence at high concentrations in other fractions that did not demonstrate significant immunosuppressive effects (e.g., fraction F2), the mycotoxin TeA was also selected to be tested in the NF-κB assay. This decision was made because TeA was the only mycotoxin present in all fractions tested, and because all fractions exhibited at least minimal suppression of the pathway. As shown in Fig. 3, while TeA did not result in any suppression of the LPS-induced activation of the NF-κB pathway up to the highest concentration tested (250 µM), the perylene quinones ALTP and ATXI suppressed the pathway in a concentration-dependent manner starting from 1 µM. Of note, the almost complete suppression of the pathway observed after exposure of cells to fraction F10 (≈ 98%) can probably be explained by the presence of ALTP and ATX-I, as they suppressed the signal by 89.54% and 23.09% when tested alone at the concentrations present in the fraction (1 µM and 2 µM, respectively) (Fig. 3a). Conversely, the reduced signal observed after incubation of cells with fraction F11 was likely a consequence of the presence of other known or unknown immunosuppressive compounds or the occurrence of combinatory effects. In fact, despite the comparable immunosuppressive effects between fractions F10 and F11, the concentrations of ALTP and ATX-I were much lower in fraction F11 (Figure S2). Since no data about the immunosuppressive properties of the other *Alternaria* mycotoxins contained in the extract are currently available in the literature, and considering that the data collected point toward the presence of yet unknown compounds with immunosuppressive properties, further studies are required to completely elucidate the compounds responsible for the immunosuppressive properties of the extract.

Besides the identification of potential immunotoxic *Alternaria* metabolites, in the present study the AlP assay was carried out to verify the possible involvement of some known *Alternaria* mycotoxins in the antiestrogenic effects of the CE. In fact, the *Alternaria* extract used in the present study was previously reported to suppress the E2-induced stimulation of the AlP activity in Ishikawa cells at concentrations ≥ 5 µg/mL (Aichinger et al. 2019). These effects were observed despite the presence of the *Alternaria* mycotoxins AOH and AME in the CE, which are known to exert estrogenic effects in the Ishikawa cell line starting from 2.5 µM (Lehmann et al. 2006; Dellafiora et al. 2018). The mycotoxin AOH was reported to mediate estrogenic effects also in the MMV-Luc estrogen-responsive cell line, with an EC_50_ ranging from 4.7 to 6.2 µM (Frizzell et al. 2013; Demaegdt et al. 2016). Despite the presence of these mycoestrogens, the onset of antiestrogenic effects is probably a consequence of their low concentration in the extract (Table S2) and the presence of other known or unknown compounds able to suppress the ER pathway. To possibly identify these compounds, the CE-fractions obtained by SFC were tested in the AlP assay. As shown in Figure S4, none of the CE-fractions induced estrogenic effects up to the highest concentration tested (1.5 µg/mL), while antiestrogenic effects were observed after incubation of cells with the highest concentration of the fractions F2, F5, and F9–11 (Fig. 4). These results confirm the presence of compounds with antiestrogenic properties in the CE. According to the LC–MS/MS results (Table 2), the tetramic acid derivative TeA was the only *Alternaria* mycotoxin detected and quantified in the antiestrogenic fraction F2. While this suggests a possible involvement of the mycotoxin in the effects induced by the fraction, its potential antiestrogenic properties were previously ruled out. In fact, TeA was found to be unable to suppress the estrogenic effects induced by 1 nM E2 in Ishikawa cells, even at concentrations of 100 µM (Crudo et al. 2022). Thus, other unknown metabolites are involved in the antiestrogenic effects induced by the fraction. Apart from fraction F2, the strongest antiestrogenic effects were observed after exposure of cells to F9, F10, and F11, which are the same fractions previously identified as exerting immunosuppressive effects (Fig. 4) and containing the mycotoxins ALTP, AOH, ATX-I, ATX-II, and TeA, among others. Considering the known estrogenic properties of AOH, the inability of TeA to suppress the pathway, and the non-commercial availability of ATX-II, the mycotoxins ALTP and ATX-I were selected for testing in the AlP assay. Notably, these mycotoxins were found to be present in significant concentrations in fraction F10, which also exhibited the strongest antiestrogenic effects (Fig. 4). As shown in Fig. 5, exposure of cells to these mycotoxins resulted in a concentration-dependent decrease in AlP activity starting from 0.4 µM and 2 µM for ALTP and ATX-I, respectively. However, ATX-I only led to minor decreases in the AlP activity compared to ALTP, which showed the ability to suppress the ER pathway by ≈ 60% at 2 µM without affecting cell viability. It must be pointed out that the antiestrogenic effects exerted by fraction F10 cannot be explained solely by the presence of ATXI and ALTP. In fact, considering the LC–MS/MS results (Table 2), cells treated with 1.5 µg/mL of fraction F10 were exposed to concentrations of ATX-I and ALTP (0.4 µM and 0.2 µM, respectively; Figure S2) lower than those found to exert antiestrogenic effects (Fig. 5). Based on this, it is plausible that the effects observed with F10, and more generally those reported for the CE by Aichinger et al. (2019), are a consequence of combinatory effects or the presence of other, yet unidentified, *Alternaria* metabolites acting as antiestrogenic compounds. Further studies are imperative to pinpoint the specific compounds or combinations responsible for the observed effects.

## Conclusion

Through a toxicity-guided fractionation of a complex *Alternaria* mycotoxin extract (CE), the present study investigates the immunomodulatory and antiestrogenic properties of this class of emerging contaminants. In particular, the results obtained clearly indicate that the *Alternaria* mycotoxin AOH plays a limited role in the immunosuppressive effects of the mycotoxin extract, suggesting the involvement of combinatory effects or the presence of other toxicologically active compounds within the extract. While the mycotoxin TeA showed ineffectiveness in exerting immunosuppressive effects, the perylene quinones ALTP and ATX-I are identified here for the first time as suppressors of the LPS-induced activation of the NF-κB pathway in THP-1 Lucia monocytes, with ALTP demonstrating significantly higher potency than ATX-I. Surprisingly, these mycotoxins also exhibit antiestrogenic effects in the Ishikawa cell line, potentially contributing to the overall antiestrogenic effects exerted by the *Alternaria* extract. However, integration of results from NF-κB gene reporter assay (immunomodulation) and alkaline phosphatase assay (antiestrogenicity) with the mycotoxin quantification performed by LC–MS/MS suggests a partial contribution of these mycotoxins to the overall immunomodulatory and antiestrogenic effects of the extract. Therefore, while this study identifies ALTP and ATX-I as novel immunosuppressive and antiestrogenic compounds from *Alternaria* fungi, enhancing our understanding of mycotoxin exposure’s health implications, it emphasizes the necessity for surveillance and further research on the occurrence and toxicological effects of *Alternaria* mycotoxins. Future investigations should prioritize elucidating the molecular mechanisms underlying the immunotoxic and endocrine-disrupting activities of these mycotoxins, as well as exploring the potential for additive or synergistic effects within complex mycotoxin mixtures. Ultimately, it is further paramount to identify the currently undisclosed active metabolites generated by this genus of molds, as it is essential for a thorough evaluation of the risks associated with consuming foods contaminated by this class of mycotoxins, to which consumers are perpetually exposed.

## Supplementary information

Below is the link to the electronic supplementary material.

Supplementary file1 (PDF 325 KB)

## Data Availability

The datasets generated during the current study are available from the corresponding author on reasonable request.

## Abbreviations

AA-III: Altenuic acid III
AlP: Alkaline phosphatase
ALS: Altenusin
ALT: Altenuene
ALTP: Alterperylenol
AME: Alternariol monomethyl ether
AOH: Alternariol
AST: Altersetin
ATX: Altertoxin
CE: Complex extract of *Alternaria* mycotoxins
CTB: CellTiter-Blue
Dexa: Dexamethasone
DMSO: Dimethyl sulfoxide
E2: Estradiol
ER: Estrogen receptor
LC–MS/MS: Liquid chromatography–tandem mass spectrometry
LPS: Lipopolysaccharide
NF-κB: Nuclear factor kappa-light-chain-enhancer of activated B cells
SE: *Alternaria* extract spiked with AOH
SFC: Supercritical fluid chromatography
STTX-III: Stemphyltoxin III
TeA: Tenuazonic acid
TEN: Tentoxin
