# Supplementary material for: Discovery of the Alternaria mycotoxins alterperylenol and altertoxin I as novel immunosuppressive and antiestrogenic compounds in vitro
Source: Arch Toxicol. 2024 Oct 2;99(1):407–21. doi: 10.1007/s00204-024-03877-1 (PMC11741999; doi:10.1007/s00204-024-03877-1)
Supplement: Supplementary file 1 — Supplementary file1 (PDF 325 KB) [file 204_2024_3877_MOESM1_ESM.pdf]

Supplementary material

Discovery of the *Alternaria* mycotoxins alterperylenol and altertoxin I as novel immunosuppressive and antiestrogenic compounds *in vitro*

Francesco Crudo[1,§], Vanessa Partsch[1,2,§], Dennis Braga[1], Ruzica Blažević[3], Judith M. Rollinger[3], Elisabeth Varga[1,4], Doris Marko[1,*]

Author affiliations:

[1] Department of Food Chemistry and Toxicology, Faculty of Chemistry, University of Vienna, Vienna, Austria;

[2]Doctoral School in Chemistry, Faculty of Chemistry, University of Vienna, Vienna, Austria;

[3]Division of Pharmacognosy, Department of Pharmaceutical Sciences, University of Vienna, Vienna, Austria;

[4]Unit Food Hygiene and Technology, Centre for Food Science and Veterinary Public Health, Clinical Department for Farm Animals and Food System Science, University of Veterinary Medicine, Vienna, Vienna, Austria;

§ These two authors contributed equally to this work

Corresponding author:
Univ. Prof. Dr. Doris Marko
Department of Food Chemistry and Toxicology, University of Vienna
Währinger Str. 38, 1090 Wien, Austria
doris.marko@univie.ac.at

Abbreviations

AA-III	Altenuic acid III
AlP	Alkaline phosphatase
ALS	Altenusin
ALT	Altenuene
ALTP	Alterperylenol
AME	Alternariol monomethyl ether
AOH	Alternariol
AST	Altersetin
ATX	Altertoxin
CE	Complex extract of Alternaria mycotoxins
CTB	CellTiter-Blue
Dexa	Dexamethasone
DMSO	Dimethyl sulfoxide
E2	Estradiol
ER	Estrogen receptor
LC-MS/MS	Liquid chromatography–tandem mass spectrometry
LPS	Lipopolysaccharide
NF-κB	Nuclear factor kappa-light-chain-enhancer of activated B cells
SE	Alternaria extract spiked with AOH
SFC	Supercritical fluid chromatography
STTX-III	Stemphyltoxin III
TeA	Tenuazonic acid
TEN	Tentoxin

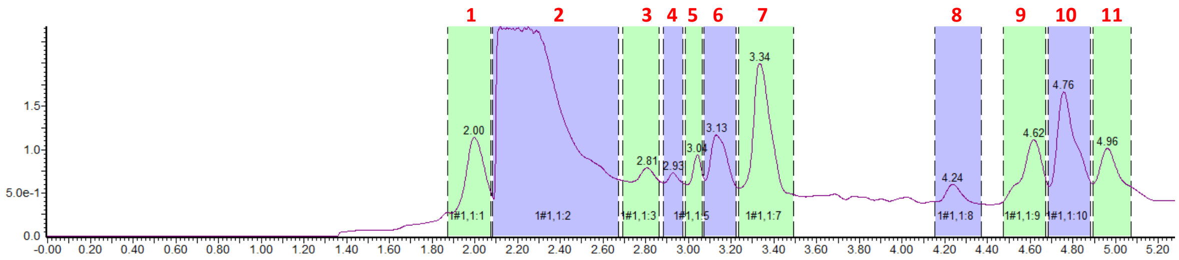

Figure S1: Representative chromatogram of the CE obtained by Supercritical Fluid Chromatography (λ=205 nm). The numbers in red and the dashed lines indicate the fractions collected and the sampling points, respectively.

Table S1: Concentration of *Alternaria* mycotoxins to which THP1-Lucia cells were exposed during treatments with the highest concentration of the tested fractions (NF-κB assay; c=7.5 µg/mL).

Fraction	Mycotoxin concentration (nM)										
	**AOH**	**AME**	**ALT**	**TeA**	**TEN**	**ATX-I**	**ATX-II**	**ALTP**	**ALS**	**AA-III**	**AST**
**F1**				68		2.7		26			
**F2**				20300							
**F3**		0.3		9030	0.25			2.3			
**F4**		0.3		7720	2.0			1.6			390
**F5**		0.3		4700	5.8	1.0		1.6			260
**F6**		0.2		6270	0.8	0.8		2.4			180
**F7**		0.2	550	6330	0.3	1.0	2.3	2.1	9.0		53
**F8**	2.7	55		5370		1.8		8.5		28	9.5
**F9**	110	6.1	8.3	4040		3.3	270	13	39	33	14
**F10**	15	2.1		2740		2000	130	1000	370		7.0
**F11**	4.9	1.6	0.004	3220		100	37	630	8.5		7.3

Abbreviations: AOH, alternariol; AME, alternariol monomethyl ether; ALT, altenuene; TeA, tenuazonic acid; TEN, tentoxin; ATX-I, altertoxin I; ATX-II, altertoxin II; ALTP, alterperylenol; ALS, altenusin; AA-III, altenuic acid III; AST, altersetin.

Table S2: Concentration of *Alternaria* mycotoxins to which Ishikawa cells were exposed during treatments with the highest concentration of the tested fractions (AlP assay; c=1.5 µg/mL).

Fraction	Mycotoxin concentration (nM)										
	**AOH**	**AME**	**ALT**	**TeA**	**TEN**	**ATX-I**	**ATX-II**	**ALTP**	**ALS**	**AA-III**	**AST**
**F1**				13.6		0.55		5.3			
**F2**				4050							
**F3**		0.05		1810	0.05			0.45			
**F4**		0.05		1540	0.38			0.32			78
**F5**		0.05		940	1.2	0.18		0.33			52
**F6**		0.05		1250	0.15	0.16		0.47			36
**F7**		0.05	110	1270	0.05	0.18	0.45	0.41	1.8		11
**F8**	0.55	11		1070		0.35		1.7		5.5	1.9
**F9**	22	1.2	1.7	807		0.65	55	2.6	7.7	6.5	2.9
**F10**	3.0	0.4		548		400	26	210	73		1.4
**F11**	0.95	0.3	0.001	643		20	7.4	130	1.7		1.5

Abbreviations: AOH, alternariol; AME, alternariol monomethyl ether; ALT, altenuene; TeA, tenuazonic acid; TEN, tentoxin; ATX-I, altertoxin I; ATX-II, altertoxin II; ALTP, alterperylenol; ALS, altenusin; AA-III, altenuic acid III; AST, altersetin.

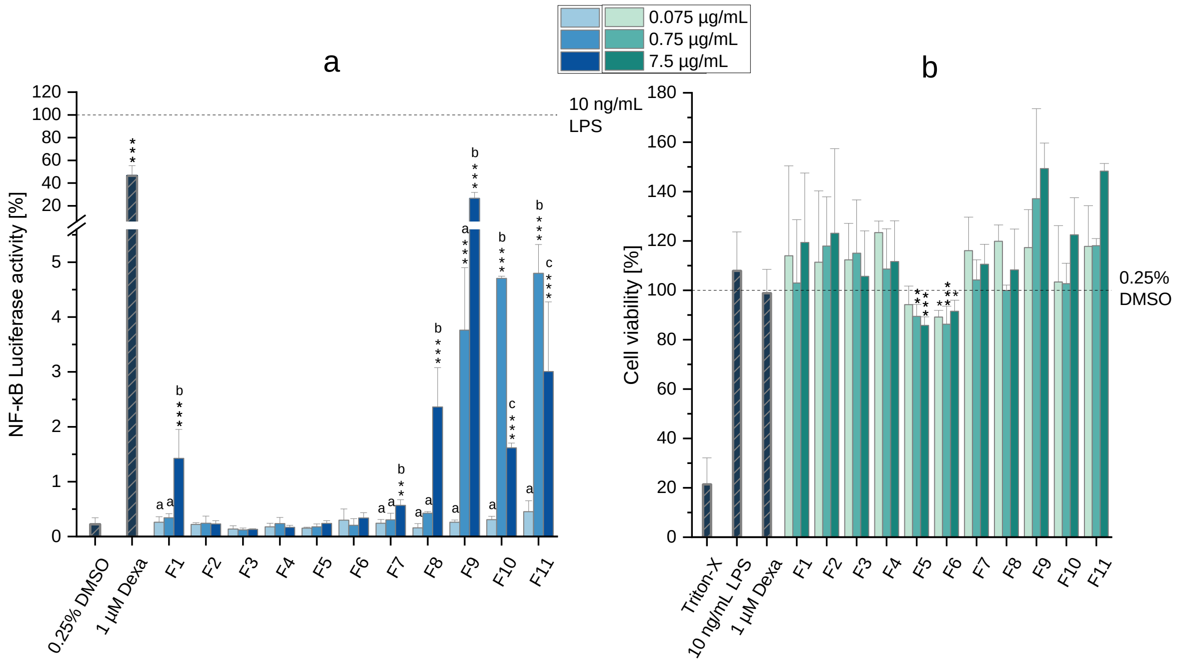

Figure S2: Effects of CE fractions on the NF-κB pathway activation (in the absence of LPS stimulation) and viability of THP-1 Lucia^{TM} monocytes. a) and b) show the results of the NF-κB and CTB assays, respectively. Results are expressed as mean + SD of at least 3 biological replicates and are normalized to the positive control (10 ng/mL LPS; in the NF-κB assay) or solvent control (0.25% DMSO; in the CTB assay). Dexamethasone (1 µM Dexa; for the NF-κB assay) and Triton-X (0.01 %; for the CTB assay) were used as controls. Statistically significant differences compared to the positive control (in the NF-κB assay) or the solvent control (in the CTB assay) were assessed by applying the Student *t*-test (*p<0.05; **p<0.01; ***p<0.001). Significant differences among the various concentrations of the fractions tested were assessed by applying the one-way ANOVA with Fisher-LSD post hoc test. Bars are marked with letters only in case of a significant difference between the groups. Different letters above the bars indicate statistically significant difference at p<0.05.

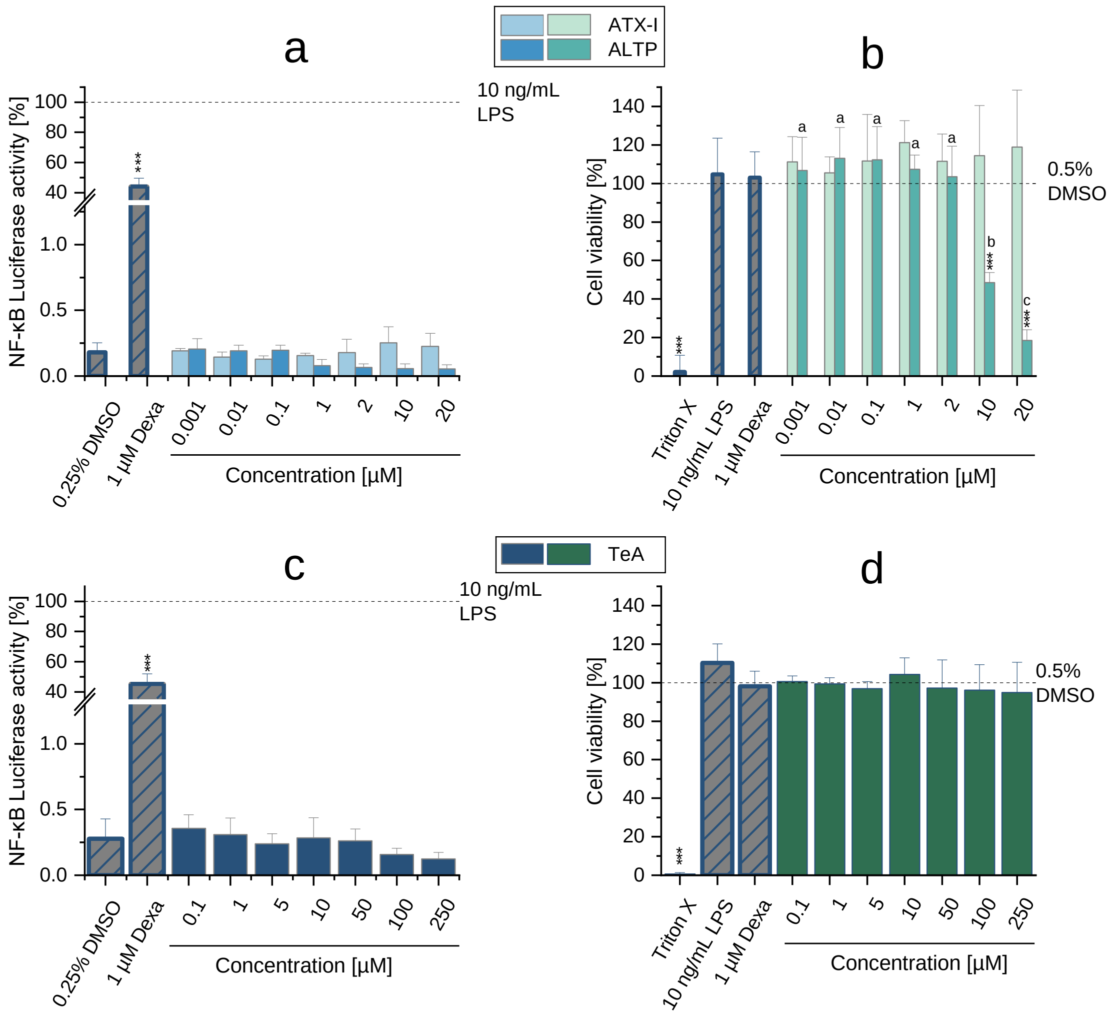

Figure S3: Effects of ATX-I, ALTP, and TeA on the NF-κB pathway activation and viability of THP-1 Lucia™ monocytes in the absence of LPS-stimulation. a) and b) show the results of the NF-κB and CTB assays (respectively) obtained from the exposure to ATX-I and ALTP, while c) and d) report the results for TeA. Results are expressed as mean + SD of at least 3 biological replicates and are normalized to the positive control (10 ng/mL LPS; in the NF-κB assay) or solvent control (0.25% DMSO; in the CTB assay). Dexamethasone (1 µM Dexa; for the NF-κB assay) and Triton-X (0.01%; for the CTB assay) were used as controls. Statistically significant differences compared to the solvent control were assessed by applying the Student *t*-test (*p<0.05; **p<0.01; ***p<0.001). Significant differences among the various concentrations of each mycotoxin were assessed by applying the one-way ANOVA with Fisher-LSD post hoc test. Bars are marked with letters only in case of significant differences among the concentrations tested. Different letters above the bars indicate statistically significant difference at p< 0.05.

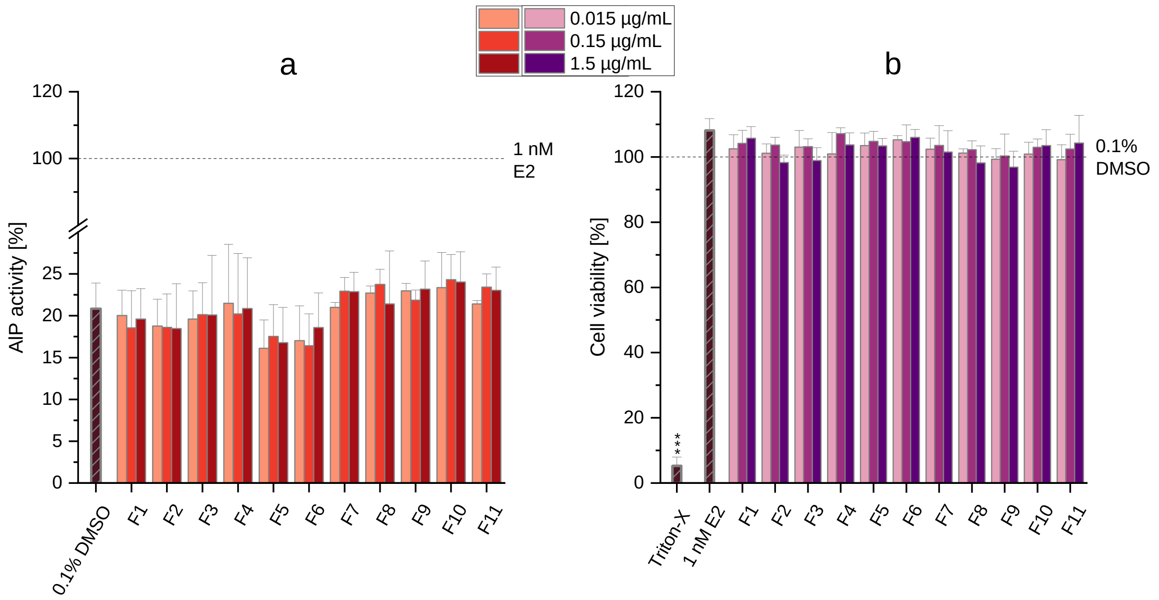

Figure S4: Estrogenic properties of CE-fractions in Ishikawa cells. a) and b) show the results of the AlP assay and CTB assay, respectively. Results are expressed as mean + SD of at least 3 biological replicates and data were normalized to the positive control (1 nM E2; in the AlP assay) or solvent control (0.1% DMSO; in the CTB assay). Triton-X (0.005 %) served as a positive control in the CTB assay.

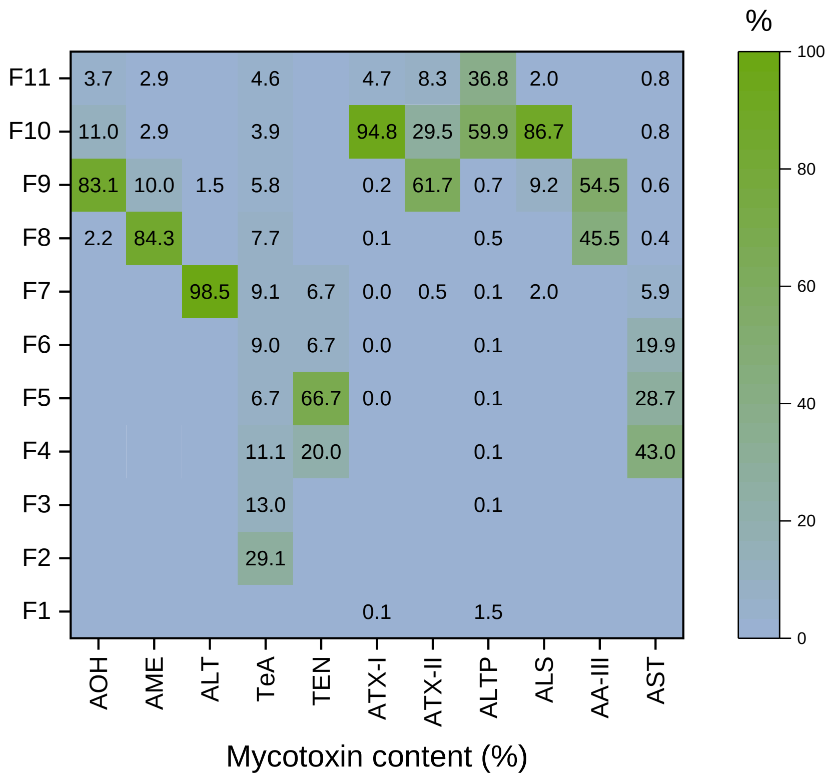

Figure S5: Heatmap showing the distribution (in %) of *Alternaria* mycotoxins in the various CE-fractions.
